# Platelet-Associated CD40/CD154 Mediates Remote Tissue Damage after Mesenteric Ischemia/Reperfusion Injury

**DOI:** 10.1371/journal.pone.0032260

**Published:** 2012-02-27

**Authors:** Peter H. Lapchak, Antonis Ioannou, Lakshmi Kannan, Poonam Rani, Jurandir J. Dalle Lucca, George C. Tsokos

**Affiliations:** 1 Rheumatology Division, Department of Medicine, Beth Israel Deaconess Medical Center and Harvard Medical School, Boston, Massachusetts, United States of America; 2 The United States Army Institute of Surgical Research, San Antonio, Texas, United States of America; French National Centre for Scientific Research, France

## Abstract

Several innate and adaptive immune cell types participate in ischemia/reperfusion induced tissue injury. Amongst them, platelets have received little attention as contributors in the process of tissue damage after ischemia reperfusion (I/R) injury. It is currently unknown whether platelets participate through the immunologically important molecules including, CD40 and when activated, CD154 (CD40L), in the pathogenesis of I/R injury. We hypothesized that constitutive expression of CD40 and activation-induced expression of CD154 on platelets mediate local mesenteric and remote lung tissue damage after I/R injury. Wild type (WT; C57BL/6J), CD40 and CD154 deficient mice underwent mesenteric ischemia for 30 minutes followed by reperfusion for 3 hours. WT mice subjected to mesenteric I/R injury displayed both local intestinal and remote lung damage. In contrast, there was significantly less intestinal damage and no remote lung injury in CD40 and CD154 deficient mice when compared to WT mice. Platelet-depleted WT mice transfused with platelets from CD40 or CD154 deficient mice failed to reconstitute remote lung damage. In contrast, when CD40 or CD154 deficient mice were transfused with WT platelets lung tissue damage was re-established. Together, these findings suggest that multiple mechanisms are involved in local and remote tissue injury and also identify platelet-expressed CD40 and/or CD154 as mediators of remote tissue damage.

## Introduction

Tissue damage following ischemia reperfusion (I/R) occurs as a consequence of deprivation of the blood flow followed by its return to the affected tissue. Re-establishment of the blood supply initiates an intense inflammatory response locally and subsequently in remote organs that involve elements of both innate and adaptive immune response [1]. Contributors to tissue damage after I/R injury include several solubles such as natural Ig [2], complement components [3], as well as cellular components including B [4], T [5], NK, NKT cells [6], and neutrophils [7]. Inhibition of complement or depletion of T or B cells has been used successfully to prevent tissue damage after I/R injury [6]. However, the contribution of platelets or platelet-derived factors in the development of tissue damage after I/R injury has not been thoroughly characterized.

Platelets typically express a pro-inflammatory phenotype and have been shown to play an important role in the onset and progression of chronic and acute inflammatory responses in rheumatoid arthritis [8], [9], systemic lupus erythematosus [10], inflammatory bowel disease [11], [12],vascular inflammation in graft rejection [13] and more recently in ischemia reperfusion injury [14]. Platelets have been also shown to activate the complement pathway and that complement components may activate platelets [15]. Thus, localized inflammation may be perpetuated in the presence of both platelets and complement components.

Activation of platelets occurs predominantly through the integrin, GPIIβ3α (CD41–CD61), which is the major platelet activation receptor. While binding of fibrinogen to GPIIβ3α leads to platelet activation [16], this activation may only be “transient” and may require additional integrins or cell surface receptors to act in synergy culminating in terminal activation [17]. Once activated, platelets express a pro-inflammatory phenotype whereby they express and release cytokines, adhesion molecules, metalloproteases, and co-stimulatory molecules such as CD154 [17].

CD154 and CD40 are important immune co-stimulatory molecules involved in isotype class switching in B cells, T cell effector function [18], and monocyte/macrophage and endothelial cell activation [19], [20], [21]. Platelets constitutively express CD40 and when activated, CD154. Engagement of platelet CD40 with CD154 has been shown to induce the release of α-granules and dense body contents; it also leads to transient cell surface expression of CD154 prior to its release into circulation [17]. Together, CD154 and CD62P expression have been shown to initiate platelet-platelet and platelet-leukocyte aggregation [22]–[24]. Thus platelet CD40/CD154 may lead to further activation of platelets, monocytes, neutrophils and endothelial cells which may culminate in remote tissue injury following mesenteric I/R. Here, we test the hypothesis that platelet expression of CD40/CD154 mediates remote tissue injury after mesenteric I/R.

We demonstrate that both CD40 and CD154 expression on platelets is important in remote lung tissue damage after mesenteric I/R injury. Our study implicates CD40/CD154 expression on platelets as important mediators of remote tissue damage.

## Materials and Methods

### Ethics Statement

All experiments were performed in accordance with the guidelines and approval of the Institutional Animal Care and Use Committee of the Beth Israel Deaconess Medical Center.

### Mice

Adult, 8 week old male C57BL/6J, *CD154^−/−^* and *CD40^−/−^* mice were obtained from The Jackson Laboratory (Bar Harbor, ME) and housed in the animal research facility at the Beth Israel Deaconess Medical Center (BIDMC) prior to experimentation. Eight to 12 week old male mice were used for all the experiments. Both male and female mice up to 24–30 weeks old were used to prepare purified platelets.

### Ischemia Reperfusion Injury Protocol

Mice were prepared for surgery after 7 days of acclimatization. They were randomly assigned to either sham or I/R groups. Anesthesia was induced with 72 mg/kg pentobarbital (Nembutal, Lundbeck Inc., Deerfield, IL) and maintained with 36 mg/kg of pentobarbital by intraperitoneal injection.

Animals were subjected to I/R as previously described [25]. Briefly, a midline laparotomy was performed then the animals were allowed a 30 minute equilibration period after which time superior mesenteric artery was identified, isolated, and then clamped for 30 minutes using a small non-traumatic micro vascular clip. The clip was removed after this ischemic phase and the intestines were allowed to reperfuse for up to 3 hours. Sham-operated group were subjected to above-described surgical intervention without artery occlusion. The laparotomy incision was sutured, the mice resuscitated with 1.0 mL pre-warmed sterile PBS subcutaneously and monitored during the reperfusion period. Body temperature was maintained at 37°C throughout the preparatory and experimental procedure. At the end of the reperfusion period, mice were euthanized by carbon dioxide asphyxiation and the tissues were harvested. A 20 cm long segments [distal to the gastro duodenal junction] of small intestinal specimens were removed, flushed with ice-cold PBS followed by ice-cold 10%phosphate-buffered formalin prior to overnight fixation in 10% phosphate-buffered formalin. Lung removal consisted of intact extraction of the bronchial tree after expansion with 200–300 µL of 10% phosphate-buffered formalin and fixed overnight in 10% phosphate-buffered formalin.

### Platelet Depletion

Two days prior to platelet transfusion and ischemia reperfusion, mice received a single intraperitoneal injection of an affinity purified endotoxin-free rabbit anti-mouse polyclonal antibody prepared with commercially available rabbit anti-mouse platelet anti-sera (Inter-Cell Technologies, Jupiter, FL) as described previously [32].

### Platelet Isolation and Transfusion

Whole blood was collected into syringes containing acid citrate dextrose by cardiac transfusion into polypropylene tubes. The blood mixture was centrifuged at room temperature and the upper phase containing platelet rich plasma was isolated, the platelets pelleted and resuspended in Tyrodes' buffer for transfusion as described previously [26]. Platelet numbers were determined using Hemavet 850 (Drew Scientific, Farmington, CT) and were adjusted to 2×l0^9^/mL. Platelet numbers were adjusted to 200 µL volumes and were transfused into platelet-depleted recipient mice ten minutes prior to initiation of the experimental midline laparotomy as described above.

### Histology and Tissue injury scoring

Formalin-fixed intestine and lung tissues were extensively washed in PBS, processed and embedded in paraffin for histological analysis. The tissues embedded in paraffin were sectioned transversely in 6 µm sections, and stained with hematoxylin and eosin. The stained sections were then subjected to histological scoring to evaluate the intestinal and lung tissue damage. All histological analysis was performed in a blinded manner.

For each intestinal section, 100 villi were graded using a 6-tiered scale as described previously [27]. Briefly, a normal appearing villus was assigned a score of 0 while villi demonstrating tip distortion were scored as 1. Villi without goblet cells and with Guggenheims' spaces were scored as 2 and villi containing patchy disruption of the epithelial cells were scored as 3. Villi demonstrating exposed, intact lamina propria and sloughing of epithelial cell were scored as 4. Villi demonstrating exuding lamina propria were assigned a score of 5, and lastly, villi with hemorrhage or denudation were scored as 6. In case of lungs, alveolar and peri-luminal injury scores for each lung section were calculated based on Cooke's method [28]. Ten to twenty fields at high power field magnification (400×) were viewed for each lung section and scored for alveolar infiltration on a 3-tiered scale. The following calculation for alveolar scores was performed as follows: a score of 0 was given when no infiltrate was present; a score of 1 was given when the infiltrate could be visualized easily only at 400×; when infiltrates were readily visible, a score of 2 was assigned; and the score for consolidation was 3. Similarly, each section was scored for peri-luminal damage (airway or blood vessel) at 100×. The calculation for peri-luminal scores was as follows: when there was no infiltrate a score of 0 was assigned; when the infiltrate was between 1 and 3 cell layers thick, the score was 1; for infiltrates ranging from 4 to 10 cells layers thick; a score of 2 was assigned; and infiltrates >10 cell layers thick were scored as 3. Based on the overall involvement of the section, a severity score was calculated: the severity score for 0–25% involvement was 1; a severity score of 2 was assigned for 25–50% involvement; and the severity score for >50% involvement was 3. For calculation of the total lung injury score, the means of alveolar and peri-luminal scores for each section for summed up and multiplied by the severity score which gave a final score ranging from 0 to 18.

### Immunohistochemistry

For immunohistochemistry, formalin-fixed paraffin sections of intestine and lung (6 µm thick) were subjected to rehydration and antigen retrieval as recommended by manufacturer (BD Biosciences, Billerica, MA). Samples were blocked in PBS+10% FCS for an hour and the sections were incubated overnight with primary antibodies. At the end of overnight incubation, the sections were washed thoroughly and were incubated with secondary antibodies for 1 h. Stained sections were developed with NovaRed (Vector Laboratories, Burlingame, CA) and counterstained with hematoxylin (Vector Laboratories, Burlingame, CA). Appropriate isotype controls were used. For immunohistochemical studies, the following reagents were used: affinity-purified rabbit polyclonal antibody, rabbit anti-mouse C3 (B-9,Santa Cruz Biotech, Santa Cruz, CA) and peroxidase-conjugated affinity-purified secondary antibodies to rabbit immunoglobulin (Jackson ImmunoResearch, West Grove, PA)

### Image Development

All images were viewed and captured using Nikon Eclipse 80i microscope and adjusted using the adjustment feature in the RGB channel using Adobe Photoshop CS2 (Adobe Systems, San Jose, CA).

### Statistical analysis

Data are presented as mean ± SEM. All data were subjected to statistical analysis using GraphPad Prism 4.0 for Windows software program (GraphPad Software, San Diego, CA). Non-parametric Mann-Whitney t test for unpaired samples was performed to compare sham controls and experimental injury data. A p≤0.05 was considered significant.

## Results

### Intestinal and lung injury are reduced after mesenteric ischemia/reperfusion in CD154^−/−^ and CD40^−/−^ mice

**Figure 1:**
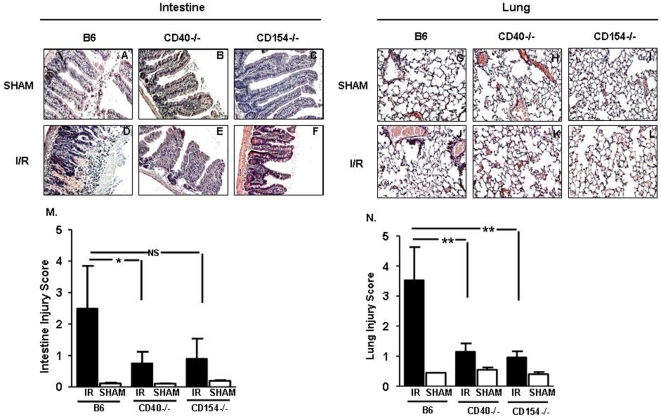
Intestinal and lung injury is reduced after mesenteric ischemia/reperfusion in *CD40^−/−^* and *CD154^−/−^* mice. Hematoxylin and eosin stained sections of mouse small intestine after 30 minutes of ischemia and 3 hours reperfusion. Images are representative of 3–4 mice per group in two experiments. (A–F) Images of intestinal villi from sham and I/R. (G–L) Images of lung from sham and I/R, platelet-deficient sham and I/R. All images shown are 200×magnification. (M, N) Injury score (mean ± SD) in intestine and lung. *p≤0.05, **p≤0.01, and ***p≤0.001 for I/R compared to sham controls.

To establish the role of CD40 and its ligand CD154, in tissue damage after mesenteric I/R, we first evaluated the levels of intestinal and lung damage in wild type (WT), *CD40^−/−^* and *CD154^−/−^* mice by histology (Figure 1). Mice lacking either CD40 or CD154 displayed a significant reduction in intestinal (Figure 1A–F) and lung damage (Figure 1G–L) compared to WT after mesenteric I/R. Cumulative data are shown in Figure 1M and Figure 1N.

### Role of CD40^−/−^ and CD154^−/−^ platelets in local and remote tissue injury after mesenteric ischemia reperfusion injury

**Figure 2:**
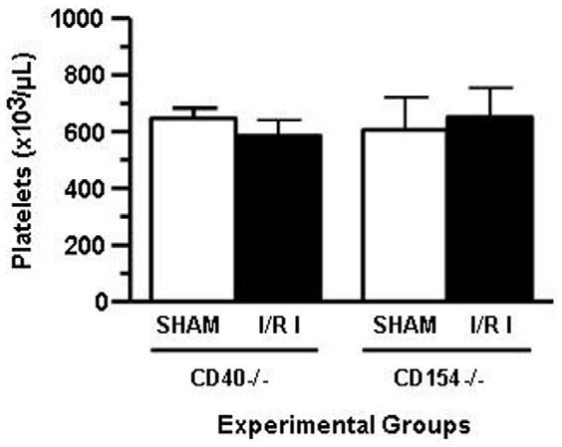
Platelets numbers are not decreased in *CD40^−/−^* and *CD154^−/−^* mice after mesenteric ischemia/reperfusion. *CD40^−/−^* and *CD154^−/−^* mice were bled before and after mesenteric I/R via cardiac puncture. Platelet numbers were determined using Hemavet 850 (Drew Scientific, Farmington, CT).

We asked whether deficiency of CD40 and CD154 affects the numbers of circulating platelets. As it can be seen in Figure 2 the absence of CD40 and CD154 did not alter the numbers of circulating platelets before and after IR. Importantly, CD40 and CD154 deficient animals have the same numbers of circulating platelets compared to WT B6 mice [14].

**Figure 3:**
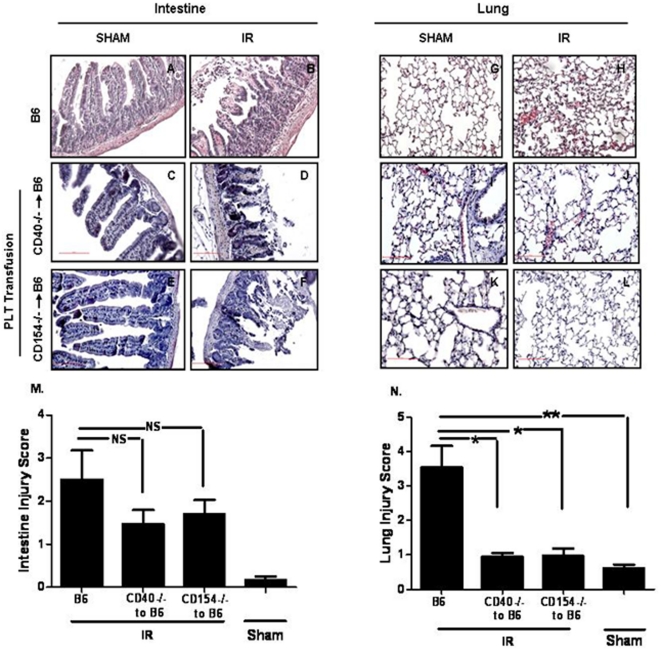
Transfusion of either *CD40^−/−^* and *CD154^−/−^* platelets protects platelet depleted B6 mice from remote lung injury but not from local intestinal injury. (A–L) Hematoxylin and eosin stained sections of mouse small intestine and lung from B6 and platelet depleted B6 mice transfused with either *CD40^−/−^* or *CD154^−/−^* platelets after 30 minutes of ischemia and 3 hours reperfusion. Images are representative of 3–4 mice per group in two experiments. All images shown are 200×magnification. (M)Intestinal injury score and (N) Lung injury score (mean ± SD). ns: not significant *p≤0.05, **p≤0.01, and ***p≤0.001 for I/R compared to sham controls.

To further evaluate the role of platelet-expressed CD40 and CD154 in local ischemia reperfusion, B6 mice were treated with an anti-platelet depleting antibody and subsequently transfused with platelets isolated either from *CD40^−/−^* or *CD154^−/−^* mice. Intestinal damage was evaluated by histology. Representative experiments are shown in Figure 3A–F and cumulative data are shown in Figure 3G. Although transfusion of CD40 or CD154 deficient platelets into platelet depleted B6 mice decreased intestinal tissue damage it did not reach statistical significance compared with control B6 mice. In contrast remote lung damage was significantly reduced in the transfused B6 mice that received platelets from *CD40−/−* and *CD154−/−*. Representative experiments are shown in Figure 3G–L and cumulative data are shown in Figure 3H. The data indicates that platelets, and especially platelet-expressed CD40 and CD154, molecules are important for remote damage after mesenteric I/R.

**Figure 4:**
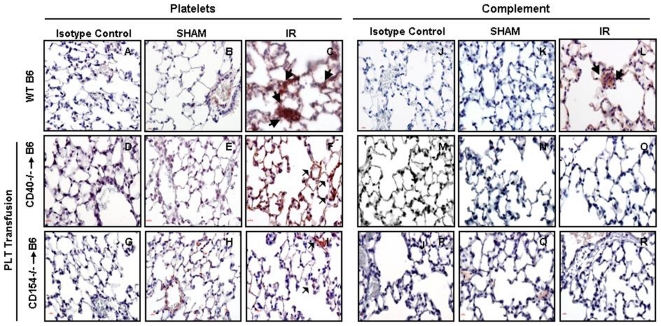
Reduced platelet and complement deposition in lungs after ischemia/reperfusion in *CD40^−/−^* and *CD154^−/−^* platelet transfused B6 mice. (A–R) Tissue sections of lung from B6 (top row) and platelet depleted B6 mice transfused with either *CD154^−/−^* (bottom row) or *CD40^−/−^* (middle row) platelets and mice after 30 minutes of mesenteric ischemia 3 hrs of reperfusion and sham controls were stained for platelets (A–I) or C3 complement factor (J–R) and counterstained with hematoxylin (blue). Images are representative of 3–4 mice per group. Large arrows: indicate areas with increased intensity of staining. Small arrows: indicate areas with reduced intensity of staining.

To further evaluate the role of platelet-expressed CD40 and CD154, platelet-transfused intestinal and lung tissues were stained for platelets and complement (Figure 4). Reduced number of platelets were observed in the lungs of mice that were first depleted of platelets and subsequently transfused with CD40 or CD154 deficient platelets (Figure 4A–I). C3 deposition followed a pattern similar to that observed with platelets (Figure 4J–R). These experiments indicate that activated platelets may drive complement pathway activation leading to remote tissue damage after mesenteric I/R.

### Local and remote injury is re-established in CD40^−/−^ and CD154^−/−^ mice transfused with B6 platelets

**Figure 5:**
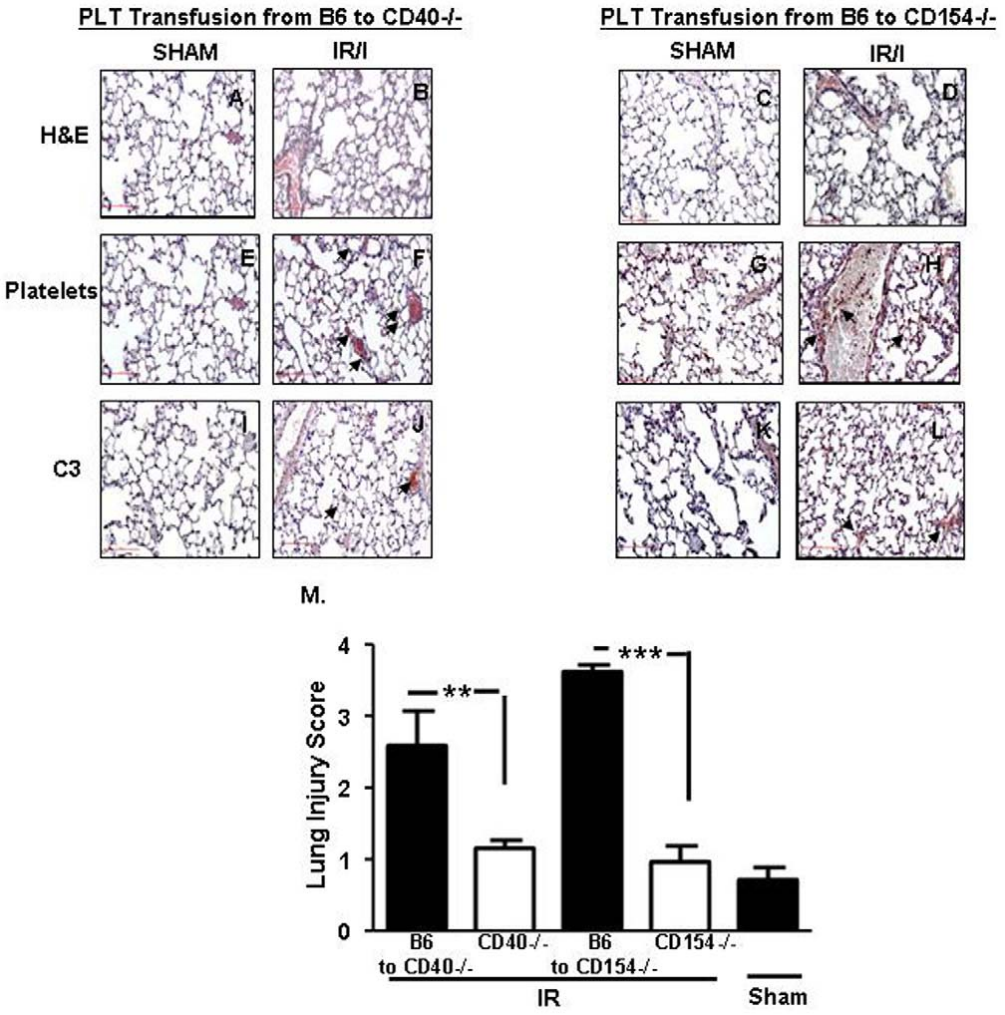
Remote lung injury is re-established in *CD154^−/−^* and *CD40^−/−^* mice transfused with B6 platelets. (A–D) Hematoxylin and eosin, (E–H) platelets and (I–L) C3 complement factor stained sections of mouse lung from *CD154^−/−^* and *CD40^−/−^* mice transfused with B6 platelets after 30 minutes of ischemia and 3 hours reperfusion. Images are representative of 3–4 mice per group in two experiments. All images shown are 200×magnification. (M)Injury score (mean ± SD) in intestine and lung. ns: not significant *p≤0.05, **p≤0.01, and ***p≤0.001 for I/R compared to sham controls. Small arrows: indicate areas of positive staining.

To confirm the role of platelet-expressed CD40 and CD154 molecules in remote lung injury, we transfused platelets from B6 mice back to platelet-depleted *CD40^−/−^* and *CD154^−/−^* mice and performed mesenteric I/R. As expected, lung injury was re-established in these mice (Representative data Figure 5A–D, cumulative data, Figure 5M). Furthermore, staining for platelets and C3 showed an increase in platelet sequestration (Figure 5E–H) and C3 deposition (Figure 5I–L) in the lung after mesenteric I/R. Together, these findings suggest that platelet-expressed CD40 and CD154 orchestrate lung damage by controlling the trafficking of platelets to the lung.

## Discussion

Experiments reported in this article grant platelet-expressed CD40/CD154 an important role in the expression of mesenteric I/R mediated remote lung injury. We first show a reduced intestinal and no lung injury in *CD40−/−* and *CD154−/−* mice after mesenteric I/R compared to WT controls. Our experiments demonstrate that transfusion of platelets from either *CD40−/−* or *CD154−/−* mice to platelet depleted B6 mice resulted in less or no lung damage compared to the control mice. In contrast, the lung tissue damage was re-established when WT platelets were transfused to platelet-depleted *CD40−/−* and *CD154−/−* mice.

Platelets contribute significantly to the expression of tissue damage in several conditions because of their pro-inflammatory nature [18], [29]–[32]. Recently, platelets have been demonstrated to contribute to the pathogenesis of a number of inflammatory diseases. Specifically, it has been demonstrated that platelets after activation by circulating immune complexes, can form aggregates with monocytes and dendritic cells and contribute to the severity of the disease in patients with systemic lupus erythematosus patients [10]. In a mouse model of rheumatoid arthritis platelets were shown to play a central role in the control of leukocyte-endothelial interactions through P-selectin and contributing to the joint damage observed in these mice [8], [33]. Moreover, increased levels of activated platelets and platelet-derived factors have also been found in patients with inflammatory bowel disease [12], [34]–[36] and with ischemic stroke [37]–[40].

CD40 is a member of the tumor necrosis factor (TNF) receptor superfamily, and is constitutively expressed on the surface of resting and activated platelets and vascular endothelial cells. Its ligand CD154, is present inside in alpha granules in resting platelets and when platelets are activated CD154 translocates to the membrane for up to 90 minutes prior to its being where it is cleaved by matrix metalloproteases and released in a soluble form. Platelets are the source of 95% of soluble CD154 in the circulation [41]. Notably recent studies have demonstrated a direct link between CD40/CD154 and complement. Specifically, C4-bining protein, an inhibitor of C3 convertase is able to interfere with the CD40/CD154 interactions by creating complexes with the CD154 and thus inhibiting the down stream signaling pathways [42]. The role of the CD40 and CD154 pathway has been extensively investigated in many diseases including cardiovascular diseases [29], [43], [44]. However, the role for platelet CD40/CD154 has not yet been evaluated in mesenteric ischemia reperfusion injury.

Mice deficient in CD40 or CD154 have altered serum immunoglobulin profile with decreased levels of IgG but increased levels of IgM [45]. In our experiments, *CD40−/−* and *CD154−/−* mice also showed decreased levels of IgG1, IgG2a, IgG2b, IgG3 but normal IgA and IgM levels (data not shown). Because natural antibodies have been implicated in the instigation of IR injury [2], [46] we considered that the decreased local and remote lung injury that we observed in *CD40−/−* and *CD154−/−* mice could be due to decreased levels of naturally occurring Ig initiating I/R injury. To test this hypothesis, we injected normal IgG into CD154KO mice prior to IR and this failed to reestablish organ damage following IR (data not shown). This information further strengthens our conclusion that it is the absence of CD40 and CD154 in platelets that accounts for the decreased IR injury in *CD40−/−* and *CD154−/−* mice.

In conclusion we have demonstrated that the expression of CD40 and/or CD154 on platelets is necessary for the expression of remote organ damage after mesenteric I/R injury. Based on these findings we suggest that the use of available compounds against platelet activation or blocking CD40–CD154 interaction may represent an effective adjuvant therapy in efforts to inhibit or even prevent the remote tissue damage.
